# Contrasting effects of pollinators on the pollination success of floral morphs of a distylous bowl-shaped flower

**DOI:** 10.1093/aob/mcaf281

**Published:** 2025-11-03

**Authors:** Javier Valverde

**Affiliations:** Departamento de Biología Vegetal y Ecología, University of Sevilla, 41012 Seville, Spain; Estación Biológica de Doñana, Consejo Superior de Investigaciones Científicas (CSIC), 41092 Seville, Spain

**Keywords:** Pollination, pollination success, flower polymorphism, distyly, *Linum narbonense*, pollen receipt, pollen tubes

## Abstract

**Background and Aims:**

In distylous species, the reciprocal arrangement of sexual organs between long-styled and short-styled individuals promotes disassortative pollen flow through a fine-tuned interaction with pollinators. Despite its evolutionary importance, deviation from this expectation due to suboptimal pollinator performance has been little studied in species with open corollas. This study addresses which insects visiting the flowers of *Linum narbonense* (Linaceae) promote or break down the expected patterns of pollination.

**Methods:**

Sixteen samplings performed in 12 populations allowed me to analyse the relationship between flower visitors and pollination performance of each floral morph. A total of 3494 stigmas were analysed to measure two components of pollination success: a quantity component that included the proportion of pollinated stigmas and pollen load on stigmas; and a quality component that included for the first time the relationship between pollen tubes and pollen grains on stigmas. Hand-pollination experiments made it possible to characterize the breeding system and to construct a null model of the pollen tube response to legitimate pollen.

**Key Results:**

Contrary to long-styled flowers, flowers with short stigmas showed wide variation in the quality of pollen receipt across sampling units, usually being lower than expected by the null model. This variation depended on the proportion of flower visits by insects from the *Usia* genus (Bombyliidae): low visitation rates by these insects were associated with lower pollen quality deposited on short stigmas.

**Conclusions:**

This study demonstrates the value of addressing the quantity and quality of pollen receipt to correlate pollination success with contemporary pollinator environment. The novel use of the relationship of pollen tube to pollen grain demonstrated the importance of *Usia* pollinators in promoting disassortative pollination in the distylous *L. narbonense*. These findings emphasize the importance of identifying which flower visitors promote the functioning of distyly or, conversely, disrupt it, biasing the functional gender of floral morphs.

## INTRODUCTION

Most flowering plant species are hermaphroditic, bearing bisexual flowers that express both female and male sexual organs (Renner, 2014). Theoretically, plants with this sexual system optimize the exploitation of pollinator participation by simultaneously benefiting the female and male reproductive functions (Charnov *et al.*, 1976; Lloyd, 1982; Christopher *et al.*, 2019). However, several aspects, such as pollinator grooming or inaccurate morphological matching with the flower, may cause pollen wastage and consequently affect the efficiency of pollen transfer (Thomson, 1986; Castellanos *et al.*, 2003; Armbruster *et al.*, 2009; reviewed in Inouye *et al.*, 1994 and Minnaar *et al.*, 2019*a*, among others), leading to an unbalanced contribution of pollinators to the reproductive success of male and female function (Wilson and Thomson, 1991; Hargreaves *et al.*, 2009; Pearson *et al.*, 2023). This bias can have important evolutionary implications for hermaphroditic plant species that present flower polymorphism. The probability of a flower visitor coming into contact with the sex organs may vary depending on the arrangement of the sex organs in each floral morph. This may result in a differential contribution to the male and female success of the flower depending on the floral morph, causing pollen flow to be more efficient in one direction (e.g. Simón-Porcar *et al.*, 2014; Minnaar *et al.*, 2019*b*). As a consequence, the functional gender of one floral morph can be biased towards a better performance as female and that of the other morph as male, directly affecting the evolutionary maintenance of flower polymorphism (Lloyd, 1979, 1980; Beach and Bawa, 1980; García-Robledo, 2008; Pannell, 2018). Addressing how pollinators differently affect the sexual performance of plant individuals is therefore a cornerstone to understanding the pollinator-mediated evolution of plant sexual systems.

Many studies reporting a bias in the sexual performance of plants involve distylous species. Distyly is a dimorphic sexual system in which individuals of two floral morphs coexist in the same population. These morphs display contrasting but reciprocal placement of male and female organs in the flower: in the short-styled morph (S-morph), stigmas are located at a lower level below the anthers, while in the long-styled morph (L-morph) this arrangement is reversed (Darwin, 1877; Barrett, 1992; Lloyd and Webb, 1992). Following Darwin’s (1877) expectations, this spatial arrangement would prevent self pollination and promote legitimate pollination between morphs through a fine-tuned morphological fit with pollinators. Pollinators would contact higher-level and lower-level sexual organs with different parts of its body, moving pollen disassortatively from S- to L-morph flowers and vice versa. In addition, this sexual system is usually accompanied by an incompatibility system that prevents fertilization by illegitimate pollen – self-pollen or outcross-pollen from the same morph (Lloyd and Webb, 1992;but see Ganders, 1975; Schou and Philipp, 1984; Zhou *et al.*, 2015; reviewed in Barrett and Cruzan, 1994). However, increasing numbers of studies have reported differences in female reproductive success between morphs (e.g. García-Robledo, 2008; Simón-Porcar *et al.*, 2014; Jiao *et al.*, 2024), which can be due to differences between morphs in pollen production (Ganders, 1979; Keller *et al.*, 2014), but can also arise from suboptimal pollinator performance. For instance, inaccurate pollinator morphological fit with flowers can lead pollen transfer to be more efficient in one direction (Lau and Bosque, 2003; Simón-Porcar *et al.*, 2014) or result in the deposition of illegitimate pollen on stigmas through self-pollination or cross-pollination with the same floral morph (Keller *et al.*, 2014). Approaching the causalities leading to such deviations from the expected patterns of pollination is important for understanding the functioning of distyly and the circumstances under which it is favoured.

Compared with distyly in species with tubular flowers, distyly in species with more open corollas is less understood and has been little explored (Björkman, 1995; Barrett, 2019; Jiao *et al.*, 2024). Open flowers pose less morphological restriction to pollinators, which usually allows visitation by a more diverse guild of animals that differ in how they fit and handle flowers (Ollerton *et al.*, 2007). Very likely, this entails higher variation in the performance of flower visitors in transferring pollen between high- or low-level organs, which hampers understanding of the functioning of distyly in such species. However, this variability offers the opportunity to explore the covariation between the pollinator assemblage and the functioning of pollination. Given the high spatio-temporal variation in the diversity and composition of pollinators visiting the flowers of generalist plant species (Gómez *et al.*, 2009; Valverde *et al.*, 2016), a covariation between pollinators and pollination success is expected. Specifically, the presence of morphologically and behaviourally well-suited pollinator species should promote pollination between floral morphs to be equally efficient in both directions, while under a suboptimal pollinator assemblage pollination success may be biased in one of the floral morphs. Analysing this relationship is therefore a first step towards identifying the most efficient pollinators, allowing us to know which pollinator assemblage promotes or hinders the disassortative pollination patterns theorized in distylous species, opening options for evolution in multiple directions.

Following the previous rationale, the main objective of this study is to advance the understanding of distyly in *Linum narbonense* (Linaceae), a species with open bowl-shaped flowers. This is accomplished by the following objectives: (1) characterize the breeding system of this species; (2) measure the diversity and composition of the assemblage of flower visitors across populations; and (3) address the pollination success of both morphs as pollen recipients and its covariation with flower visitors. The genus *Linus* comprises several distylous species that alternate throughout the clade with homostylous species and species with other related types of sex organ distribution (Ruiz-Martín *et al.*, 2018; Maguilla *et al.*, 2021). Several observational studies on the visitation rate and behaviour of *Linum* spp. flower visitors suggest that insects of the genus *Usia* (Bombyliidae, Diptera) are true pollinators and may have played an important role as selective agents in the evolution of the sexual systems of this clade (Armbruster *et al.*, 2006; Ruiz-Martín *et al.*, 2018; Foroozani *et al.*, 2023; Pérez-Barrales and Armbruster, 2023). For example, Armbruster *et al.* (2006) described a fine-tubed morphological fit of *Usia* species with the sexual organs of *L. suffruticosum*. However, to date no study has directly linked flower visitors to pollen transfer efficiency in a *Linum* species, this study being the first to do so.

Studies addressing pollen transfer efficiency in distylous species usually take advantage of the usual morphological differences between pollen grains from each morph to address the amount of legitimate versus illegitimate pollination (e.g. Ornduff, 1966; Björkman, 1995; Jacquemyn *et al.*, 2018; Furtado *et al.*, 2021). However, unlike other polymorphic *Linum* species (Laibach, 1928; Dulberger, 1974; Şafak Odabaşi, 2022), pollen grains from both floral morphs of *L. narbonense* are alike in morphology and size (see Materials and methods). To overcome this problem, this study adapts the method proposed by Alonso *et al.* (2012) to calculate the quality of pollen deposition through the relationship between deposited pollen grains and developed pollen tubes and uses hand-pollination experiments as a null model to interpret and compare the results with the expected outcomes under legitimate pollination. So far, this is the first study to use this method on distylous species and amongst the few to explore the relationship between flower visitors and the performance of distyly in plant species with non-tubular corollas.

## MATERIALS AND METHODS

### Study species and sampled populations

**Fig. 1.**
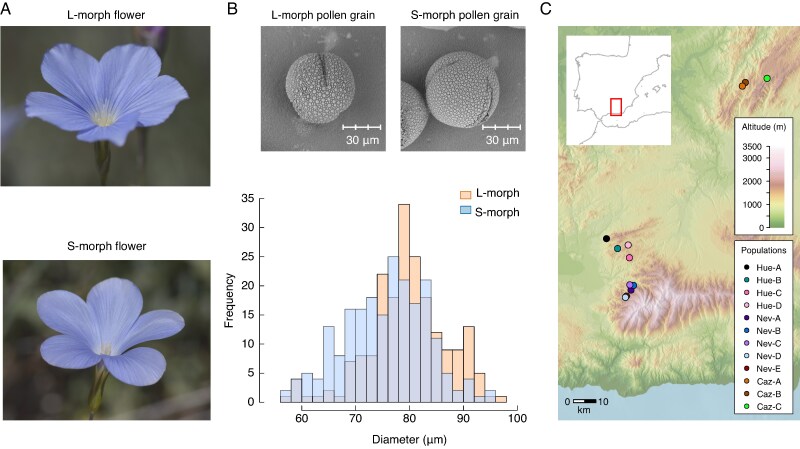
Study system. (A) L- and S-morph flowers of *Linum narbonense*. (B) Pollen sizes. Microscopy pictures of dry pollen were acquired using a Phenom Pro G6 Desktop SEM (Thermo Scientific). The histogram depicts the size distribution of 500 hydrated pollen grains per floral morph from three flowers. (C) Map showing the locations of sampled populations.

*Linum narbonense* (Linaceae) is a distylous perennial herb (Fig. 1A) that inhabit calcareous shrublands and open woodlands in the western Mediterranean region. Flowers have five bright blue free petals that form an open bowl-shaped corolla ∼33.8 mm wide (range 26.3–41.6 mm) × 11.8 mm deep (range 9.2–15.2 mm) (data from 55 plants). Flowers bear five free stamens and five styles fused at the base with lengths of 16.5 ± 1.5 and 10.5 ± 1.0 mm for L-morph and S-morph flowers, respectively (data from 313 flowers). The ovary has ten ovules, each on a single ovary chamber. S-morph flowers produce ∼1.2 times more pollen grains than L-morph flowers (estimated pollen grains per anther 1723 ± 80 and 2069 ± 219 for L-morph and S-morph, respectively; see Supplementary Information). Pollen grains from both morphs are similar in size (L-morph, 80.0 ± 7.3 μm; S-morph, 75.6 ± 7.8 μm), being indistinguishable under the microscope (Fig. 1B).

This study was performed on 12 populations along three mountain ranges from the south-east Iberian Peninsula: Sierra Nevada (populations Nev_A to E), Sierra de Huétor (Hue_A to D) and Sierra de Cazorla (Caz_A to C; Supplementary Data Table S1, Fig. 1C). Populations spanned areas ranging from 120 to 1500 m^2^ at altitudes from 1135 to 1875 m a.s.l. (Supplementary Data Table S1).

### Hand pollination experiments

The breeding system of *L. narbonense* was studied through two hand-pollination experiments. Randomly selected closed flowers from different individuals were isolated using paper tea bags. Once opened, one of the following treatments was applied. (1) Legitimate pollination: addition of pollen from three random individuals from the opposite morph. (2) Illegitimate pollination: addition of pollen from three individuals from the same morph. (3) Self-pollination: addition of pollen from the same flower. (4) Control: flower kept isolated from flower visitors. Flowers were tagged and bagged to prevent further visits.

To evaluate the response in seed production, this experiment was conducted in 2019 on 182 flowers from the Nev_A population. For each treatment, the probability of a flower producing a fruit and a seed were calculated by recording flower fruiting and seed production (Supplementary Data Fig. S1A). The probability of a flower producing a seedling was calculated by sowing all retrieved seeds (126 and 114 from L- and S-morph flowers) in seedling trays and by recording their germination success for 15 d. Seedling trays were daily watered and randomly re-orientated.

To evaluate the response in pollen tube germination, hand-pollination treatments were applied in 2021 on 102 flowers from the Caz_D population and the legitimate pollination treatment was again applied in 2023 on 66 flowers from the Nev_A population (Supplementary Data Table S1). For each treatment, the incidence of stigmas with any pollen tube was addressed. Pistils were collected after 4 d and stored in 70 % ethanol. This time period is enough for pollen tubes to reach the ovary (pers. obs.). Pistils were then stained using aniline blue: 1 m KOH at 65 °C for 30 min for softening, and 1 % aniline blue decolourized in 1 m solution of K_3_PO_4_·H_2_O at 65 °C for 45 min for staining. The presence of pollen grains and pollen tubes was assessed under a microscope (Nikon Eclipse 80i) equipped with a UV light source (CoolLED pE-300). In this species, softening with KOH makes the stigmatic tissue transparent enough to visualize all pollen grains, including those behind the visible stigmatic surface. To obtain comparable estimates between treatments, pollen tubes were observed right below the stigma (Supplementary Data Fig. S1B), minimizing potential effects derived from differences in style length and the time elapsed after pollination.

### Field study

In 2020, 2021 and 2023, the 12 populations were sampled one to three times, resulting in a total of 16 sampling units (referred to here as samples). Samples taken in the same population and year were spaced a minimum of 2 weeks apart to ensure data independence. The assemblage of flower visitors of *L. narbonense* was characterized for each sample by four to six censuses depending on the diversity of flower visitors. A census consisted of 10 min of observation along a previously defined 20 × 1 m linear transect. Any interaction of an insect with a flower of *L. narbonense* was recorded and photographed. Pictures and some captured specimens allowed the identification of flower visitors to the genus level. Censuses were spaced half an hour apart and were performed during the hours of peak pollinator activity (1100–1600 h) and in direct sunlight to improve the consistency and comparability between samplings (Mahon and Hodge, 2022). Average flower visitation rate and the relative visitation rate of each flower visitor to each morph were calculated for each sampling. Finally, the morph ratio of *L. narbonense* plants in each population was assessed by counting the number of flowering plants of each morph along the previous transects.

To characterize the pollination success associated with each sample, a minimum of 20 flowers per morph were randomly collected from different individuals. Only flowers with falling petals were collected. This ensured that the flowers were already pollinated or wilted, so that the data obtained reflected the result of the pollination process throughout the flower's lifespan. Pistils were immediately stored in individual vials containing ethanol 70 %. The number of pollen grains on stigmas and the number of pollen tubes were measured using the aniline blue method previously described.

### Data analysis

#### Breeding system

Parameters obtained from the hand-pollination experiments were analysed using logistic regressions. Treatments showing complete separation (those in which all values were 0 or 1) were discarded from the analyses. The proportion of stigmas showing any pollen tube was analysed using a mixed-effects model in which flower identity was a random factor. *Post hoc* paired comparisons were carried out using Tukey’s range test (R package multcomp; Hothorn *et al.*, 2008).

#### Flower visitors

For each sample, sampling coverage (proportion of the estimated number of species; Chao and Jost, 2012) was calculated using the R package iNEXT (Hsieh *et al.*, 2016). Then, flower visitor richness (the number of insect genera) was estimated for each sampling using rarefaction or extrapolation at an equal sample size of 20 pollinator visits. This value is close to the average sample size found (35.8 ± 28.9) and allows a reliable extrapolation of the three samplings with lower sampling coverages (Hue-C-1, 18; Nev-A-1, 11; Nev-A-3, 5). The assemblage of flower visitors of *L. narbonense* was explored using rank-abundance plots. Finally, to determine the preferences of the most frequent flower visitors for any floral morph, binomial tests were performed using data on the visitation frequency.

#### Quantity component of pollination success

For each sample, the pollination success of floral morphs was measured by means of two components: a quantity component and a quality component of pollen receipt. The quantity component measures the incidence and amount of conspecific pollen on sampled stigmas and was characterized using two parameters: (1) proportion of pollinated stigmas: proportion of stigmas with any conspecific pollen; and (2) pollen load: the average number of conspecific pollen grains on pollinated stigmas.

To test whether floral morphs differed in each parameter at each sample, individual generalized models were constructed per sample using floral morph as explanatory variable. For the percentage of pollinated stigmas, models followed a binomial distribution and used the bias-reduction method developed by Firth (1993) to deal with cases with complete separation (brglm R package v.0.7.2; Kosmidis, 2021). Overall differences across samples were addressed through additional models that considered all data and that included floral morph and sample identity as interacting variables. For the pollen load, models followed a Poisson distribution and included flower as a random factor (lme4 R package v.1.1-37; Bates *et al.*, 2015). To test for overall differences across samples, additional models were constructed that nested flower identity within sample in the random structure.

#### Quality component of pollination success

**Fig. 2.**
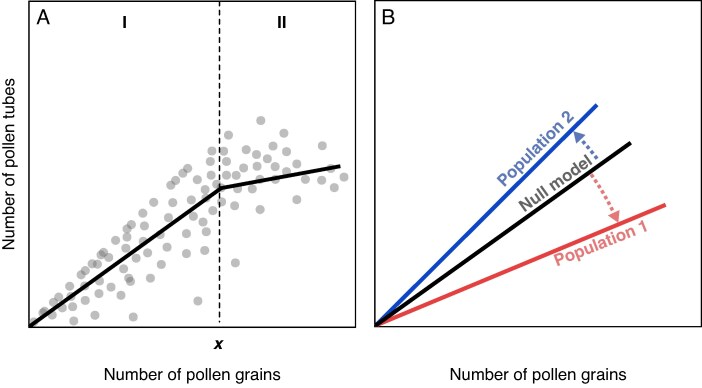
Quality component of pollination success. (A) Dose–response of pollen tubes to pollen load on a stigma modelled by a broken linear function. The quality component (b1) is given by the slope of the first segment of the function (I), which describes the increase in pollen tubes up to a saturation point (*x*), after which the slope of response of pollen tubes is smoother (II). (B) The quality component obtained from hand-pollinated flowers can serve as a null model (black line) to evaluate the pollination quality of natural populations. Lower slopes (population 1, red line) will indicate a lower pollination quality, here due to a higher proportion of illegitimate pollen grains from flowers of the same morph or from self-pollination. Higher slopes (population 2, blue line) will indicate a higher pollination quality.

The quality component is a proxy of the proportion of legitimate pollen grains on stigmas and was measured as the slope of response of pollen tubes to pollen load. This component of pollination success was measured for each sampling but also using data from hand pollination experiments to obtain a null model (see below). Following the methodology of Alonso *et al.* (2012), the dose–response followed by the number of growing pollen tubes to pollen load (Brink, 1924; Cruzan, 1986) was modelled for each sampling using piecewise regressions (segmented R package v.1.4-0; Muggeo, 2017). The resulting broken linear function deals with the sudden change in the relationship slope due to saturation (Fig. 2A). The slope of the first segment (b1) was then used as a measure of the quality component. To calculate b1, independent zero-intercept linear mixed effects models were fitted for each sampling using data from single stigmas (Alonso *et al.*, 2012). Floral morph was included as interacting covariate and flower identity as random factor. The existence of a saturation breakpoint was then evaluated for each model using Davies’ test at a *P*-value <0.01. If the test was significant, a piecewise regression was constructed from the initial linear mixed effects model, otherwise the latter was maintained. In any case the resulting models were visually examined.

The latter methodology was also used to obtain a null model depicting the expected response in number of pollen tubes under legitimate pollination (pollen grains from the opposite morph) in L- and S-morph stigmas. Null models were constructed by pooling data from the legitimate pollination treatment in Nev_A and Caz_D populations, given the lack of difference in such response between populations (see Supplementary Information).

The quality component was compared between floral morphs and with the null models at each sampling (Fig. 2B) using mixed-effects models. Models included flower as a random factor and considered data from the first segment (b1) in cases where saturation was present or all data in cases where no saturation was detected. *Post hoc* paired comparisons allowed comparison of slopes between cases. To address overall trends across samplings, an additional similar model was constructed that nested flower ID within sampling in the random structure. For simplicity, this model did not include the null model.

#### Effects of flower visitors on pollination success

To explore which flower visitors may be correlated with the parameters of pollination success in each morph, a multivariate analysis approach using redundancy analysis was used. This constrained ordination method allowed exploration of the combination of predictor variables (i.e. each flower visitor visitation rate) that best explained the variation in a set of response variables (i.e. the parameters describing the quantity and quality components of pollination success; see above). For this analysis, the Hellinger-transformed visitation rate of each flower visitor was used to minimize the effects of different total visitation rates, while parameters of pollination success were centred and standardized (Legendre and Gallagher, 2001). Next, a forward selection procedure based on the Akaike information criterion was used to find the best linear model for each of the quantity and quality parameters of pollination success. Model selection considered the relative visitation rate of any flower visitor showing any evidence of strong correlation in the redundancy analysis, flower visitor richness and the proportion of flowers from the opposite morph as potential explanatory variables.

## RESULTS

### Breeding system

*Linum narbonense* showed a sporophytic incompatibility system. Legitimate treatment resulted in 87 and 97 % of flowers producing a fruit in L- and S-morph flowers (Supplementary Data Table S2) with no differences between floral morphs (*|z|* = 1.35, *P* = 1; Supplementary Data Tables S2 and S3). Only three fruits were produced after illegitimate pollination (L-morph, 1; S-morph, 2) and no fruit was retrieved after self pollination and in control flowers. The probability of producing a seed after legitimate pollination was higher in S- (0.67) than in L-morph flowers (0.50; *|z|* = 3.69, *P* < 0.001; Supplementary Data Tables S2–S4). Similarly, the probability of producing a seedling was higher in S- (0.52) than in L-morph flowers (0.40; *|z|* = 2.56, *P =* 0.010). From the 850 stigmas used in hand-pollination treatments, only those pollinated with legitimate pollen showed any pollen tube growing (probability of stigmas with pollen tubes = 0.77 and 0.74 for L- and S-morph, respectively; Supplementary Data Table S5) and no differences were found between morphs (|*z*| = 0.210, *P* = 1; Supplementary Data Tables S6 and S7).

### Flower visitors

**Fig. 3.**
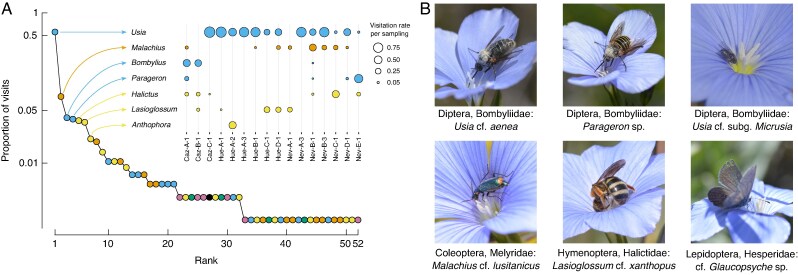
Flower visitors. (A) Rank-abundance plot of proportion of visits performed by each insect genus in this study. Inset plot depicts for each sampling the visitation rate of the most important flower visitors, performing 80 % of all visits. (B) Pictures of some of the flower visitors.

A total of 572 insect–flower interactions were recorded in 98 censuses. This resulted in an average sampling coverage of 0.83 (range 0.58–1; Supplementary Data Table S8). Flower visitor richness (number of insect genera) at a sample size of 20 contacts to flowers varied from 2 to 14 across samples. Flower visitors belonged to 57 genera and 32 families (Supplementary Data Table S9), although 80 % of flower visits were performed by the following genera: *Usia* (55.7 %, Diptera), *Malachius* (7.7 %, Coleoptera), *Bombylius* (4.0 %), *Parageron* (3.8 %, Diptera), *Halictus* (3.7 %, Hymenoptera), *Lasioglossum* (3.5 %, Hymenoptera) and *Anthophora* (2.1 %, Hymenoptera; Fig. 3). Based on the observed visits to each morph, these flower visitors showed no preference for any morph (range of probabilities of a visit being to a L-morph flower, 0.35–0.57; *P* = 0.210–1; Supplementary Data Table S10).

### Quantity component of pollination success

**Fig. 4.**
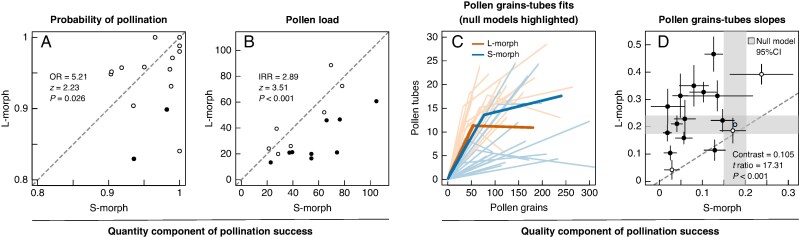
Pollination success. (A, B) Quantity component: biplots showing for each sampling (A) the probability of pollination, i.e. the proportion of stigmas with conspecific pollen, and (B) the mean pollen loads on pollinated stigmas found in each floral morph. (C, D) Quality component. (C) Segmented fits of the response of pollen tubes to pollen load in each sample and floral morph. Thicker lines represent null models. (D) Biplot of the slopes of the previous relationship (first segment). Shaded areas indicate the 95 % CI of slopes of null models of L- and S-morph flowers. Filled symbols in plots (A), (B) and (D) depict significant differences between morphs.

A total of 3494 stigmas collected from 740 flowers were analysed. The probability that a stigma was pollinated ranged from 0.83 to 1.00 in the samplings. S-morph stigmas showed higher odds of being pollinated than L-morph stigmas in 10 out of the 16 samplings, although this difference was significant only in Caz-A-1 and Nev-A-1 samplings (odds ratio S-/L-morph = 5.20 and 2.90 for the two samplings, respectively; Fig. 4A; Supplementary Data Table S11). When pooling all data, S-morph stigmas showed significantly higher odds than L-morph stigmas across samplings (odds ratio = 5.21; *z* = 2.23; *P* = 0.026). As for the pollen load, the average number of conspecific pollen grains on a stigma varied between 13 and 14. S-morph stigmas received on average more pollen than L-morph flowers in 13 samplings, for nine of which this difference was significant, with S-morph stigmas receiving 1.68 to 4 times more pollen than L-morph stigmas (Fig. 4B; Supplementary Data Table S11). This outperformance of S-morph stigmas in pollen load was also significant across samplings (incidence rate ratio S-/L-morph = 2.89; *z* = 3.51; *P* < 0.001).

### Quality component of pollination success

The response in pollen tubes to pollen load saturated in 14 of the 34 cases, including both null models (Fig. 4C; Supplementary Data Table S12). Comparisons between null models showed that under legitimate hand pollination the quality component (response in pollen tubes to pollen load before the saturation point) was similar between morphs (b1 = 0.21 and 0.18 for L- and S- stigmas respectively; *t* ratio = 1.27; *P* = 0.206). Under open pollination, the quality component varied between 0.03 and 0.47 across samplings. L-morph stigmas showed significantly higher slopes of response in 13 samplings (L–S-morph difference in estimated slopes = 0.05–0.31; Fig. 4D). When compared with their corresponding null models, slopes of L-morph stigmas did not show any general trend, while slopes of S-morph stigmas were consistently lower than expected under legitimate pollination (Fig. 4D; Supplementary Data Table S12). Overall, L-morph stigmas outperformed S-morph stigmas in the response of pollen tubes to pollen load (L–S-morph estimated difference in slope = 0.105, *t* ratio = 17.31, *P* < 0.001).

### Effects of flower visitors on pollination success

**Fig. 5.**
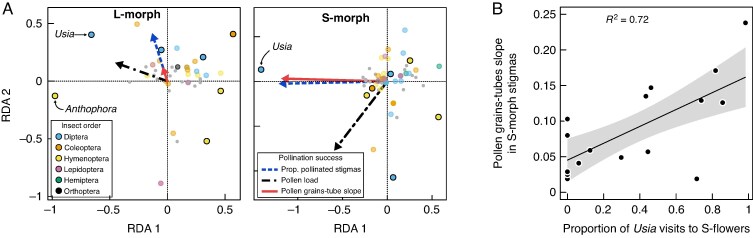
Effects of flower visitors on pollination success. (A) Redundancy analysis (RDA) ordination plots illustrating each parameter of pollination success and the assemblage of flower visitors found in each floral morph. The most important flower visitors present in a minimum of four samplings are highlighted. (B) Partial regression of the effect of the proportion of visits performed by *Usia* spp. on the pollination quality of S-morph flowers.

Redundancy analyses explained 15.2 and 27.8 % of the between-sample variation in visitation rates of flower visitors to L- and S-morph flowers. The biplot showed a dispersed distribution of visits to L-morph flowers, with some genera (e.g. *Usia*, *Anthophora*; Fig. 5A) deviating slightly from the main cluster of flower visitors, while on S-morph flowers the data showed a strong deviation of *Usia* visits from the main cluster of flower visitors and suggested an association with both the proportion of pollinated stigmas and the slope of pollen tube response to pollen load (Fig. 5A). Models of the pollination quantity parameters showed that the proportion of pollinated L-morph stigmas depended positively and significantly on the proportion of flowers of the opposite morph at a site (Table 1), while for S-morph stigmas this parameter showed a negative relationship with flower visitor richness. Pollen load did not show a significant relationship with any of the explanatory variables (Table 1). Finally, as for pollination quality, the slope of pollen tube response to pollen load in S-morph flowers depended on the proportion of visits by *Usia* (Table 1). Specifically, a higher proportion of visits by these insects increased the slope of response of pollen tubes to pollen load to that expected by the null model (Fig. 5B).

**Table 1.** Summary of the correlates of pollination success parameters. The table shows the results of the best linear model adjusted for each parameter and floral morph. Standardised estimates and 95 % CI are shown together with the test statistic and *P*-value. *P*-values in bold denote significance at α = 0.05.

		L-morph			S-morph		
Parameter	Variable	Standardised estimate	*t*	*P*	Standardised estimate	*t*	*P*
Percentage of pollinated stigmas							
	Intercept	0.00 (−0.43 to 0.43)	9.80	**<0.01**	0.00 (−0.42 to 0.42)	18.48	**<0**.**01**
	Richness				−0.67 (−1.13 to −0.22)	−3.21	**<0**.**01**
	Proportion opposite morph	0.57 (0.13–1.02)	2.80	**0**.**02**	0.36 (−0.11 to 0.84)	1.67	0.12
	*Malachius*				0.44 (−0.02–0.89)	2.10	0.06
	*Lasioglossum*	−0.32 (−0.77 to 0.12)	−1.57	0.14			
Pollen load							
	Intercept	0.00 (−0.46 to 0.46)	−0.32	0.76	0.00 (−0.53 to 0.53)	8.89	**<0**.**01**
	Richness	−0.34 (−0.84 to 0.16)	−1.48	0.16			
	Proportion opposite morph	0.42 (−0.08 to 0.92)	−1.83	0.09			
Slope of response of pollen tubes to pollen load							
	Intercept	0.00 (−0.53 to 0.53)	8.55	**<0.01**	0.00 (−0.32 to 0.32)	3.76	**<0**.**01**
	Proportion opposite morph				−0.51 (−0.85 to −0.16)	−3.16	**<0**.**01**
	*Usia*				0.67 (0.33–1.00)	4.35	**<0.01**
	*Malachius*				−0.23 (−0.58 to 0.12)	−1.43	0.179

## DISCUSSION

This study shows that flower visitors can have contrasting effects on the pollination success of floral morphs of *L. narbonense*, a distylous plant with a bowl-shaped flower. By analysing the number of pollen grains on stigmas and its relationship with the number of pollen tubes, the results show that the quality of pollen receipt is similar between floral morphs when insects of the genus *Usia* are the main pollinators, while their absence causes a lower pollination quality in the S-morph flowers in comparison to L-morph flowers. These findings demonstrate the value of analysing the relationship between the number of pollen tubes and the number of pollen grains on stigmas together with hand-pollination experiments to understand the efficiency of pollen transfer in dimorphic floral systems, especially those that lack evident pollen dimorphism. The use of this method is discussed below, followed by a discussion on the variation found in pollination success and the effects of flower visitors on such variation.

### Measurement of pollination success

The analysis of stigmatic pollen load and its relationship to pollen tube success has been useful in studies addressing the pollination success of natural populations (Alonso *et al.*, 2013; Castilla *et al.*, 2016; Albor *et al.*, 2024) and under controlled conditions (Arceo-Gómez and Ashman, 2011). Compared with other estimates based on fruit production or seed set, this approximation directly links pollen deposition, which depends on the diversity and frequency of pollinator visits, with fertilization, which is determined by the viability and compatibility of deposited pollen loads (Harder *et al.*, 2016). This permits the exploration of hypotheses on the effect of pollinators on pollination success (e.g. Zhu *et al.*, 2015) while avoiding variation among plants in their ability to produce seeds, frequently also related to resource availability (Mitchell, 1997; P. E. Gibbs, 2014).

This study demonstrates that analysing the pollination–fertilization interface is also a good avenue to address pollination success in distylous species. The classical approach of directly determining the amount of legitimate and illegitimate pollen on stigmas (e.g. Jiao *et al.*, 2024) is not feasible in distylous species that lack dimorphic pollen features (but see the method proposed by Pailler *et al.*, 2002). Alternatively, in species with diallelic sporophytic incompatibility, the fact that illegitimate pollen does not germinate on stigmas enables the use of the ratio of pollen tubes to pollen grains as a measure of the quality of the pollen load (Liu *et al.*, 2024). The present study goes a step further by using segmented models to deal with the density-dependent and -independent factors that shape the dose–response relationship followed by pollen tube success (Alonso *et al.*, 2013; Harder *et al.*, 2016). On the one hand, this method allows modelling the sudden change in slope that reflects negative density-dependent effects arising from competition with other conspecific grains present on the stigma (Brink, 1924; Harder *et al.*, 2016). On the other hand, the slope of the relationship before such change reflects density-independent factors affecting the quality of pollen receipt, such as the degree of genetic incompatibility (Hiscock and Allen, 2008; P. E. Gibbs, 2014). In the case at hand, given that illegitimate pollen does not produce any pollen tubes, the first slope should reflect the proportion of legitimate (compatible) pollen on stigmas and therefore the quality of pollen receipt.

In addition, the present study incorporates hand-pollination experiments to better interpret the results. Firstly, it makes it possible to characterize the incompatibility system of the study species and therefore to interpret the pollen grain–tube relationship as detailed above. Specifically, illegitimate pollen did not produce any seed and did not germinate, demonstrating a sporophytic diallelic incompatibility in *L. narbonense*, in line with most distylous plants (Barrett and Shore, 2008) and other dimorphic *Linum* species (Murray, 1986). Moreover, the lack of pollen found in bagged stigmas evidenced the absence of autonomous self-pollination. Secondly, legitimate hand-pollinations were used to construct a null model describing the expected slope of response in pollen tube number under legitimate pollen. As will be seen below, these two pieces of information were crucial to interpreting the variation across samplings in the quantity and quality components of pollination success in the light of the data on visitation and behaviour of flower visitors. Further studies should consider the use of this approximation in combination with hand-pollination experiments to explore the importance of pollinators in the pollen transfer and evolution of other species with different floral polymorphisms or sexual systems, such as enantiostyly, tristyly or gynodioecy.

### Pollination success in *Linum narbonense*

In most samplings, S-morph stigmas received more conspecific pollen, despite the fact that flowers of this morph have shorter stigmas than L-morph flowers (2.03 ± 0.40 versus 2.52 ± 0.60 mm; estimates obtained from 127 and 186 flowers, respectively). Yet, the quality of this pollen was lower than that of pollen deposited on L-morph stigmas. This is evidenced by the slopes of the relationship between the number of pollen grains and the number of pollen tubes found in the S-morph stigmas, which were lower than those found in the L-morph stigmas in 13 of the 16 samplings. On top of that the comparison with the respective null model also shows lower slopes than expected under legitimate pollination. This latter result indicates that the lower pollination success found in S-morph flowers in comparison with that in L-morph flowers is due to the presence of illegitimate pollen, which may result either from outcrossing pollination with flowers of the same morph and/or from pollinator-assisted self-pollination.

Differences between floral morphs in pollination and reproductive success are common among distylous plants (e.g. Ornelas *et al.*, 2004; Simón-Porcar *et al.*, 2014; Deschepper *et al.*, 2018) and have been usually attributed to suboptimal pollinators that show morphological mismatches with flowers or unsuitable modes of flower handling (Zhu *et al.*, 2015). In the more studied tubular-shaped distylous species the explanation is straightforward, given that flower architecture restricts access to a few animals with a fine morphological fit and enforces a precise location of pollen on their bodies, improving pollen transfer and reducing pollen wastage (Armbruster *et al.*, 2009; Armbruster and Muchhala, 2009; Stewart *et al.*, 2022). For distylous species this promotes an accurate segregation of pollen on the pollinators’ bodies and consequently disassortative pollination (Ganders, 1979; Keller *et al.*, 2014; Deschepper *et al.*, 2018). Thus, pollinators with non-matching morphological traits may cause a bias in pollination success between morphs by different means. For instance, studies show that pollinators with a shorter proboscis only participate in pollen transfer between high organs (Arroyo and Dafni, 1995; Simón-Pórcar *et al.*, 2014; Deschepper *et al.*, 2018). Also, suboptimal pollinators may smear pollen on other parts of their bodies during probing, which can lead to pollen wastage or to the deposition of illegitimate pollen on stigmas (Armbruster *et al.*, 2009; Brys and Jacquemyn, 2015).

In contrast, in species with more open flowers, such as those of *L. narbonense*, flower architecture permits access to a more diverse pollinator assemblage (e.g. Nikkeshi *et al.*, 2015) that shows different modes of flower handling (Ollerton *et al.*, 2007). Certainly, this hinders understanding of the mechanisms of legitimate pollination in these species but also the identification of the multiple circumstances under which pollen wastage and illegitimate pollination occur. To advance the understanding of the functioning of distyly in *L. narbonense*, this study takes the first steps to identify the association between pollinators and pollination success in each floral morph.

### Flower visitors and their correlation with pollination success in *L. narbonense*

A total of 53 insect genera from 33 families and 6 orders visited the flowers of *L. narbonense* in the 16 samplings. These values demonstrate a generalist pollination system if compared with other Mediterranean species in the study area. For example, estimates of flower visitor diversity for 221 plant species in the Sierra de Cazorla showed an average richness of 17 genera, 11 families and 3 orders (Herrera, 2021), although the larger area covered should be considered here. Despite the relatively high diversity of flower visitors found, most visits were made by several species of the genus *Usia*, in agreement with previous observations in populations from southern France (Du Merle, 1971) and Spain (Pérez-Barrales and Armbruster, 2023).

This study suggests that pollination of *L. narbonense* is optimal when *Usia* insects are the main pollinators. Seventy-two per cent of the variance in pollination quality found in the S-morph stigmas was explained by a positive relationship with the visitation rate of these insects. The model predicts that when *Usia* performs nearly 100 % of all visits pollination quality is similar to that expected under legitimate pollination. On the contrary, the variation in pollination quality in L-morph stigmas showed no correlation with the assemblage of flower visitors. Together with field observations on flower handling, these results indicate that although ecologically generalist, *L. narbonense* may be phenotypically specialized to *Usia* bee-flies. Similar to what has been previously observed in *L. suffruticosum* (Armbruster *et al.*, 2006; Pérez-Barrales and Armbruster, 2023), *Usia* bee-flies land on petals to crawl down the floral tube to reach the nectaries at the bottom of the flower. The interaction with the larger specimens results in segregation of pollen grains on their dorsal region, placing pollen from L-morph flowers on their head and pollen from S-morph flowers on their thorax (Fig. 3B), which very likely results in disassortative pollen transfer between morphs. However, depending on the size of the gap between the upper-level organs and the petal, smaller specimens of *Usia*, mostly from the *Micrusia* subgenus, may not be able to fully contact these sexual organs and not participate in pollen transport from L-morph to S-morph flowers. It is important to note that these morphological and behavioural characteristics are shared by pollinators of the genus *Parageron* (Bombyliidae; Fig. 3B), which likely are equally effective and have the same size trade-offs as those discussed above. The effect of *Usia* (and probably *Parageron*) in the pollination success of S-morph flowers adds to previous studies that point to an important role of these pollinators in the evolution of the genus *Linum*. First, there is a geographical coincidence between the diversification areas of *Usia* (D. Gibbs, 2011, 2014) and *Linum* species (McDill *et al.*, 2009; Dressler *et al.*, 2014; Maguilla *et al.*, 2021). Second, *Usia* is amongst the most frequent pollinator in several Mediterranean *Linum* species (Du Merle and Mazet, 1978; Johnson and Dafni, 1998; Armbruster *et al.*, 2006; Foroozani *et al.*, 2023; Pérez-Barrales and Armbruster, 2023), including *L. narbonense*. Finally, a previous study by Armbruster *et al.* (2006) described an exclusive type of distyly in *L. suffruticosum* that segregates pollen placement between dorsal and ventral parts of the body of these insects.

On the other hand, the results suggest that the differences found between floral morphs in pollination success may be due to inadequate flower handling by insects not belonging to the Usiini tribe. For instance, I observed that small bees foraging for pollen curled and moved around the anthers, probably transferring self pollen to stigmas, specially in S-morph flowers. Moreover, models showed that under higher pollinator diversity a lower number of S-morph stigmas were pollinated. This could be explained by the fact that some of the insects visiting the flowers of *L. narbonense* are pollen consumers and do not make contact with flower sexual organs. For instance, although *Malachius* beetles were the second most frequent flower visitors, these insects consume pollen, probably contributing to pollen loss (Fig. 3B). Long-tongued insects that forage from above the flowers, such as butterflies and *Bombylius* species, extract nectar with little or no contact with the sexual organs, given the low morphological constraints of the open corolla (Fig. 3B). Single-visit experiments should be performed to support these hypotheses, but also to shed light on other relationships that are more difficult to interpret, such as the negative relationship found between the proportion of opposite-morph flowers and pollination quality in S-morph flowers.

This study adds to others that demonstrate the importance of the performance of flower visitors as pollinators in the evolution of distyly (e.g. Zhu *et al.*, 2015). Whereas the action of well-suited efficient pollinators results in the disassortative pollination expected in a distylous system, different pollination success between floral morphs promoted by suboptimal pollinators may eventually lead to variations in functional gender (Robertson, 1892; Horovitz, 1978; Lloyd, 1980; Zhu *et al.*, 2015). Pollinator-mediated biases in the reproductive success of floral morphs where one morph performs better in its female function and the other morph in its male function have been proposed as a prelude to the evolution of dioecy (separate sexes) from hermaphroditism (Lloyd, 1979; Beach and Bawa, 1980). Following this reasoning, *Usia* flies may be favouring the maintenance of distyly in *L. narbonense*, while other, less efficient pollinators may be exerting different selective pressures towards the breakdown of this sexual system. However, the number of pollen tubes exceeded that of available ovules per flower, and thus it is unlikely that female performance is biased. Moreover, in species with intra-morph incompatibility, deviations from the expected pollination patterns may have negligible impact on mating patterns (Zhou *et al.*, 2015). Yet, this study indicates that male performance in S-morph flowers may be less efficient because of pollen wastage through self-pollen deposition or illegitimate pollination. Given that male reproductive success is more limited by the availability of mating partners (Bateman, 1948; Moore and Pannell, 2011), pollen wastage in one of the morphs may affect sexual selection and therefore the evolution of distyly. Further studies in this and other plant species should address pollinator efficiency in both female and male sexual functions, ideally throughout the flowering period, to provide insight into the role of pollinators in the evolution of polymorphic sexual systems.

## Supplementary Material

mcaf281_Supplementary_Data

## Data Availability

The dataset will be available upon request.

## SUPPLEMENTARY DATA

Supplementary data are available at *Annals of Botany* online and consist of the following. Table S1: information on the sampled populations. Tables S2–S7: statistics on the breeding system. Tables S8–S10: information on the diversity of flower visitors and floral morph preference. Table S11: measurements of quantity components of pollination success. Table S12: measurements of quality components of pollination success. Figure S1: pictures of viable and aborted seeds and pollen tubes and a plot of the breeding system. Supplementary Information: methods used for estimation of pollen production and comparison of pollen tube response to legitimate pollination between populations Nev_A and Caz_D.
