# Supplementary material for: Contrasting effects of pollinators on the pollination success of floral morphs of a distylous bowl-shaped flower
Source: Ann Bot. 2025 Nov 3;137(3):713–24. doi: 10.1093/aob/mcaf281 (PMC12933682; doi:10.1093/aob/mcaf281)
Supplement: mcaf281_Supplementary_Data [file mcaf281_supplementary_data.zip › Supplementary_Figures.pdf]

Contrasting effects of pollinators on the pollination success of floral morphs of a distylous bowl-shaped flower

Supplementary Figures

a) b)

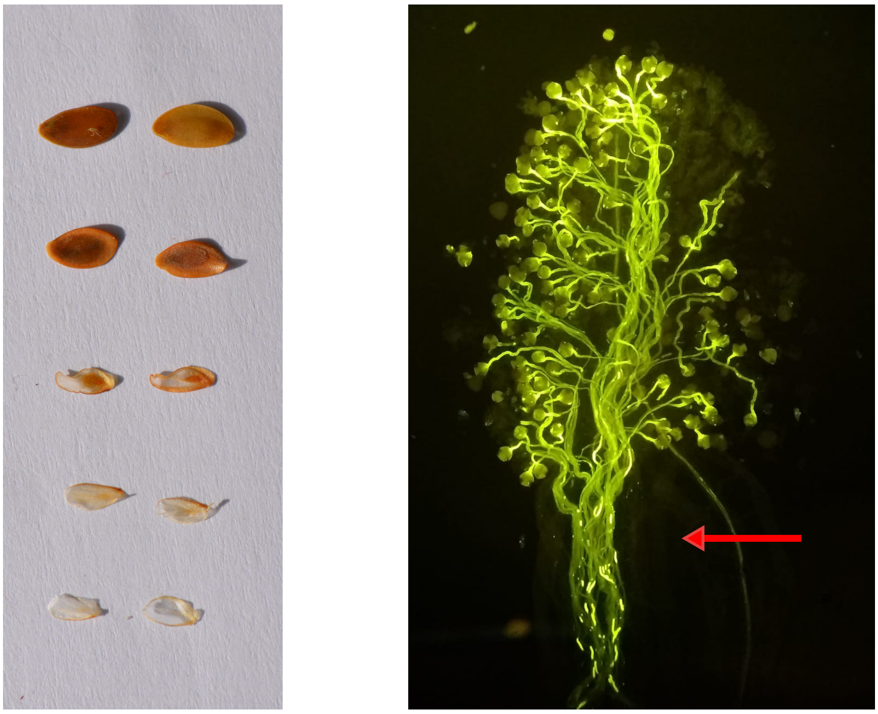

Figure S1.- a) Distinction between viable and non viable (aborted) seeds. The two seeds at the top of the photo are viable seeds. They are distinguishable by being swollen and brown to dark brown in colour. Non-viable seeds are easily distinguished by not being swollen and by their whitish/transparent colour. b) Fluorescence microscopy image showing pollen tubes stained with aniline blue. The upper part corresponds to the stigma where pollen grains can be distinguished. The arrow indicates the beginning of the style where the pollen tubes were counted in both morphs .

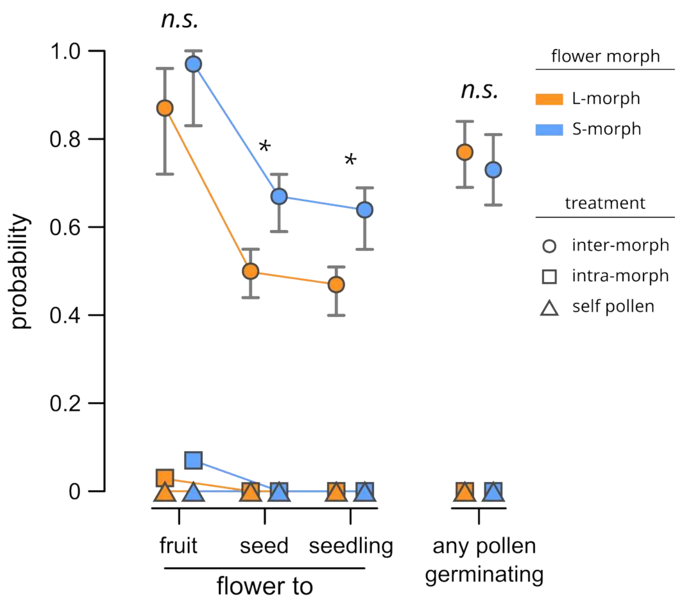

Figure S2.- Breeding system of *Linum narbonense*. The plot depicts the results from the two hand-pollination experiments. On the left, the probabilities of a hand-pollinated flower to produce a fruit, a seed and a seedling. On the rightmost the probability of finding any pollen tube at a stigma. Error bars indicate 95% confidence intervals after exact binomial tests. For simplicity, the results from the facilitated and autonomous self-pollination treatments are represented using a unique symbol.
