# Supplementary material for: Contrasting effects of pollinators on the pollination success of floral morphs of a distylous bowl-shaped flower
Source: Ann Bot. 2025 Nov 3;137(3):713–24. doi: 10.1093/aob/mcaf281 (PMC12933682; doi:10.1093/aob/mcaf281)

Contrasting effects of pollinators on the pollination success of floral morphs of a distylous bowl-shaped flower

Supplementary Information

Estimation of pollen production

Pollen production of each floral morph was estimated from 10 flowers per morph collected in the HP population. An anther was extracted from each flower and placed in a vial with 200µL of basic fuchsin solution (0.001%), which was exposed to an ultrasonic bath for 20 minutes to extract and disaggregate pollen grains. The pollen present in each vial was estimated from a minimum of five 20µL samples which were photographed under a Leica MZ-125 stereo microscope. This allowed to count pollen grains using the ImageJ software (see figures a to c below). Given the differences between anther in the variance of and to estimate an average pollen production per anther. Given the differences between anthers in the standard deviation of pollen counts (see figure c), pollen production per anther and morph was calculated using an inverse-weighted mean, giving more weight to individual means with smaller standard errors:

$$\mu = \frac{\sum \bar{x}_i / \sigma^2}{\sum 1/\sigma^2}$$

Figures.- a) Picture of a 20µL sample of stained pollen grains. b) Pollen grains identified in the previous picture by ImageJ software following a selection based on colour, size (greater than 40 µm) and circularity (greater than 0.30). c) Count data.

a)

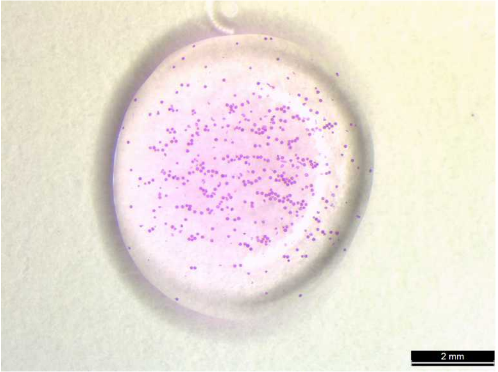

b)

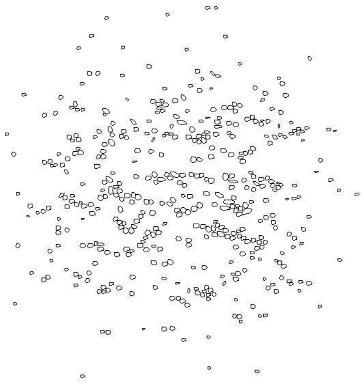

c)

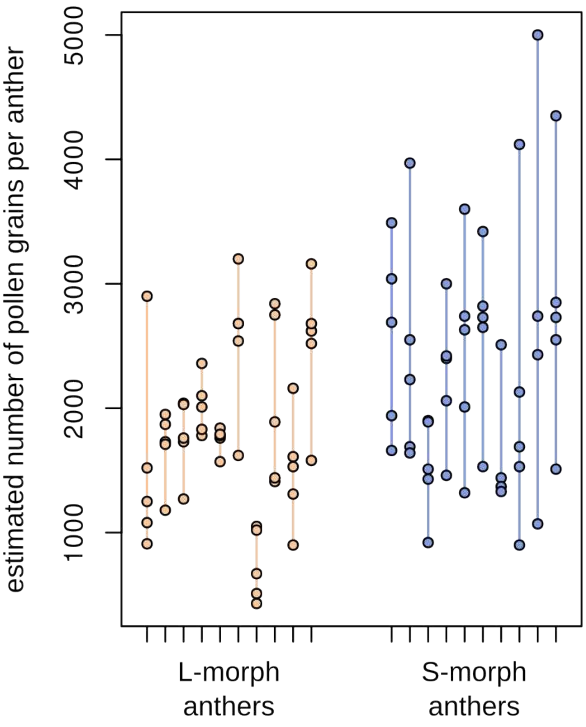

Comparison of pollen tube response to legitimate pollination between Nev_A and Caz_D populations.

The response of L- and S-morph stigmas to legitimate pollination was compared between the two populations in which the hand pollination experiment was conducted. For each floral morph, a zero-intercept mixed linear effects model of the response of pollen tubes to legitimate pollen grains was constructed. Population (Nev_A or Caz_D) was included as an interaction factor. Post-hoc comparisons between populations were performed using Tukey's range test (see main text). The results showed that the response of pollen tubes to legitimate pollen grains was similar between populations.

Table.- Models and post-hoc comparisons of the pollen tube response to legitimate pollen grains. For each floral morph, model estimates of the response at each population are shown. Post-hoc comparisons between populations are also shown.

					Post-hoc comparison (Nev_A - Caz_D)		
Floral morph	population	estimates	t	p	coefficients	t ratio	p
L-morph							
	Nev-A	0.114 [0.090–0.140]	9.375	**<0.001**	0.027 ± 0.017	1.527	0.13
	Caz_D	0.088 [0.060–0.110]	7.215	**<0.001**			
S-morph							
	Nev-A	0.128 [0.110–0.150]	13.530	**<0.001**	0.026 ± 0.014	1.850	0.07
	Caz_D	0.102 [0.080–0.120]	10.440	**<0.001**			

Figure.- Model fits.

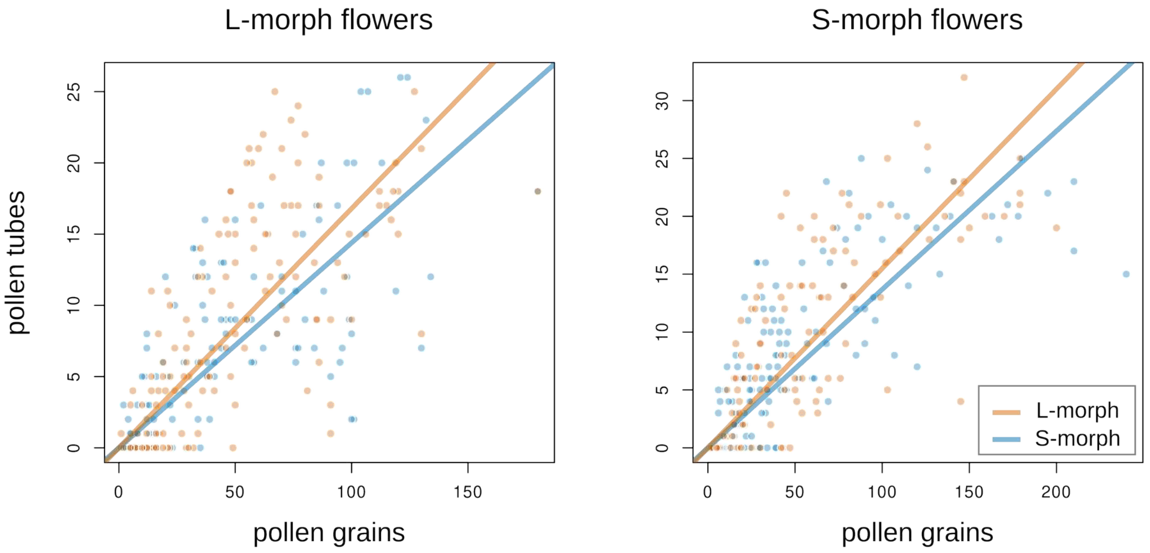

Supplement: mcaf281_Supplementary_Data [file mcaf281_supplementary_data.zip › Supplementary_Information.pdf]
