# Supplementary material for: Contrasting effects of pollinators on the pollination success of floral morphs of a distylous bowl-shaped flower
Source: Ann Bot. 2025 Nov 3;137(3):713–24. doi: 10.1093/aob/mcaf281 (PMC12933682; doi:10.1093/aob/mcaf281)
Supplement: mcaf281_Supplementary_Data [file mcaf281_supplementary_data.zip › Supplementary_Tables_Reviewed.pdf]

Contrasting effects of pollinators on the pollination success of floral morphs of a distylous bowl-shaped flower

Supplementary tables

Table S1.- Sampled populations. The table depicts the spatial location of the sampled populations and the number of samplings (samples) performed per year.

population	location	coordinates	altitude (m a.s.l.)	Pop. size (m^2)	habitat	number of samples		
						2020	2021	2023
Sierra de Cazorla								
Caz_A	Puente de Guadahornillos	37°55'42"N 2°52'14"W	1610	300	Shrubland		1	
Caz_B	Raso del Madrigal	37°54'51"N 2°53'08"W	1600	200	Shrubland		1	
Caz_C	Puerto Lezar	37°56'40"N 2°45'46"W	1640	290	Mixed shrubland-pasture		1	
Sierra de Huétor								
Hue_A	Collado Cruz de la Atalaya	37°18'51"N 3°33'35"W	1135	860	Open olm oak woodland	2	1	
Hue_B	Calarillo de las Minas	37°16'31"N 3°30'17"W	1400	600	Open olm oak woodland	1		
Hue_C	Barranco del Puerto Blanco	37°14'21"N 3°26'46"W	1365	600	Pine woodland	1		
Hue_D	Puerto de la Mora	37°17'21"N 3° 27' 8"W	1348	1500	Open mixed woodland			1
Sierra Nevada								
Nev_A	Hoya de Pedraza	37°06'43"N 3°26'17"W	1875	1400*	Shrubland	3		
Nev_B	El Dornajo	37°07'49"N 3°25'32"W	1853	720	Shrubland	2		
Nev_C	Barranco de la Víboras	37°07'57"N 3°26'38"W	1595	120	Open mixed woodland	1		
Nev_D	La Cortijuela	37°05'03"N 3°27'53"W	1810	640	Open pine woodland	1		
Nev_E	Collado de Matas verdes	37°05'16"N 3°27'40"W	1875	700	Open pine woodland	1		

* This population may be larger given the presence of sparse isolated plants beyond the main population cluster.

SUPPLEMENTARY TABLES FOR THE BREEDING SYSTEM

Table S2.- Breeding system. Descriptive statistics of probabilities of a flower to produce a fruit and of a flower to produce a seed and a seedling. The latter two are relativised to the number of ovules per flower (10). *Num*. depicts the ratio of success. Probabilities are averaged per flower and show a 95% confidence interval.

morph	treatment	flower to fruit		ovule to seed		ovule to seedling	
		num.	probability	num.	probability	num.	probability
L	legitimate	33/38	0.87 [0.72–0.96]	157/319	0.50 [0.44–0.55]	110/285	0.40 [0.33–0.45]
	illegitimate	1/36	0.03	0/10	0	0/0	0
	self-pollination	0/10	0	0/0	0	0/0	0
	control	0/9	0	0/0	0	0/0	0
S	legitimate	30/31	0.97[0.83–1]	145/222	0.67 [0.57–0.72]	96/190	0.52 [0.43–0.58]
	illegitimate	2/30	0.06	0/20	0	0/0	0
	self-pollination	0/6	0	0/0	0	0/0	0
	control	0/9	0	0/0	0	0/0	0

Table S3.- Breeding system. Models of the probability of a flower in producing a fruit, a seed and a seedling.

predictor	odds ratio	CI	p	R^2	No. obs.
Flower to fruit probability				0.758	135
intercept	6.60	2.82 – 19.28	<0.001		
L-morph illegitimate	0	0.00 – 0.03	<0.001		
S-morph legitimate	4.55	0.68 – 89.74	0.178		
S-morph illegitimate	0.01	0.00 – 0.05	<0.001		
Flower to seed probability					56
intercept	0.97	0.78 – 1.21	0.780		
S-morph legitimate	1.94	1.37 – 2.77	<0.001		
Flower to seedling probability					53
intercept	0.63	0.49 – 0.80	<0.001		
S-morph legitimate	1.62	1.12 – 2.36	0.010		

Models used: *glm(response.variable ~ flower.morph, data = data, family = binomial).*

Table S4.- Breeding system. Post-hoc pairwise comparisons between treatments in the probability of a flower in producing a fruit, a seed and a seedling (see Table S3)

comparison		coefficients	z	p
Flower to fruit probability				
S-morph legitimate	S-morph illegitimate	6.04 ± 1.253	4.82	8.5×10^{-6}
	L-morph legitimate	1.514 ± 1.124	1.35	1
	L-morph illegitimate	6.957 ± 1.436	4.85	7.6×10^{-6}
L-morph legitimate	L-morph illegitimate	5.442 ± 1.122	4.85	7.4×10^{-6}
	S-morph illegitimate	4.526 ± 0.875	5.17	1.4×10^{-6}
S-morph illegitimate	L-morph illegitimate	0.916 ± 1.251	0.73	1
Flower to seed probability				

S-morph legitimate	L-morph legitimate	0.66 ± 0.18	3.69	2.25×10^{-4}
Flower to seedling probability				
S-morph legitimate	L-morph legitimate	0.49 ± 0.19	2.56	0.0104

Table S5.- Breeding system. Descriptive statistics of the presence of pollen tubes on pollinated stigmas. The table shows the ratio of stigmas found with any pollen tube. Probabilities are averaged per flower and show a 95% CI.

morph	treatment	analysed flowers	stigmas with any pollen tube	
			number	probability
L-morph	legitimate	62	207/270	0.77 [0.71–0.82]
	illegitimate	16	0/79	0
S-morph	legitimate	62	206/278	0.74 [0.69–0.79]
	illegitimate	12	4/40	0

Table S6.- Breeding system. Models of the proportion of stigmas showing any pollen tube.

predictor	odds ratio	CI	p
Fixed effects			
intercept	882.18	83.22 – 9351.79	<0.001
S-morph legitimate	1.25	0.16 – 9.96	0.833
S-morph illegitimate	0.00	0.00 – 0.00	<0.001
Random effects			
σ^2 = 3.29	ICC = 0.96		
τ_{00} flor = 70.23	N_{flor} = 136		

Number of observations = 573; Marginal R^2 = 0.113; Conditional R^2 = 0.960.
Model used: *glmer(presence.of.tubes ~ hand.pol.treatment + (1|flower),*
data = data, family="binomial")

Table S7.- Breeding system. Post-hoc pairwise comparisons between treatments in the probability of any pollen tube growing on stigmas. See Table S4 for model parameters.

comparison		coefficients	z	p
S-morph legitimate	S-morph Ilegitimate	15.058 ± 3.028	4.974	1.97×10^{-6}
	L-morph legitimate	0.222 ± 1.059	0.210	1
L-morph legitimate	S-morph Ilegitimate	14.835 ± 3.051	4.862	3.48×10^{-6}

SUPPLEMENTARY TABLES FOR THE FLOWER VISITORS

Table S8.- Sample coverage and richness of flower visitors. For each sampling, observed and estimated genera richness at an even sample size of 20 flower visits.

sampling	observed flower contacts	sample coverage	richness		
			observed	sample size-based estimate	coverage-based estimate
Sierra de Cazorla					
Caz-A-1	28	0.78	10	8 (R)	8 (R)
Caz-B-1	27	0.84	9	7 (R)	8 (R)
Caz-C-1	131	0.96	5	1 (R)	2 (R)
Sierra de Huétor					
Hue-A-1	46	0.91	5	2 (R)	3 (R)
Hue-A-2	27	0.96	3	2 (R)	3 (R)
Hue-A-3	32	0.97	3	1 (R)	3 (R)
Hue-B-1	34	0.85	7	2 (R)	5 (R)
Hue-C-1	18	0.80	7	5 (R)	7 (E)
Hue-D-1	45	0.87	9	5 (R)	7 (R)
Sierra Nevada					
Nev-A-1	11	0.97	7	6 (R)	8 (E)
Nev-A-3	5	1.00	2	2 (R)	2 (E)
Nev-B-1	60	0.82	12	4 (R)	6 (R)
Nev-B-3	40	0.95	4	1 (R)	3 (R)
Nev-C-1	19	0.59	10	14 (E)	10 (E)
Nev-D-1	22	0.58	12	14 (E)	11 (R)
Nev-E-1	27	0.84	7	4 (R)	6 (R)

(R) = estimations performed using rarefaction
(E) = estimations performed using extrapolation

Table S9.- Flower visitors. List of insect genera recorded in the study:

Order	Family	Genus
Coleoptera	Bruchidae	*Bruchidae*
	Buprestidae	*Anthaxia*
	Chrysomelidae	*Cryptocephalus*
	Dasytidae	*Dasytes*
	Meloidae	*Meloidea*
	Meloidae	*Mylabris*
	Melyridae	*Malachius*
	Melyridae	“Melyridae 1”
	Melyridae	*Psilothrix*
	Nitidulidae	“Nitidulidae 1”
	Prionoceridae	*Lobonyx*
	Tenebrionidae	*Proctenius*
	Tenebrionidae	“Tenebrionidae 1”
	Unknown	“Coleoptera 1”
Diptera	Anthomyiidae	“Anthomyiidae 1”
	Bibionidae	*Bibio*

Order	Family	Genus
Hemiptera	Miridae	*Calocoris*
	Miridae	*Hadrodemus*
	Pentatomidae	*Pentatomidae*
	Unknown	“Hemiptera 1”
Hymenoptera	Andrenidae	Andrena
	Apidae	Anthophora
	Apidae	Apis
	Apidae	Ceratina
	Apidae	Eucera
	Chrysididae	Chrysididae
	Formicidae	Formicidae
	Halictidae	Halictus
	Halictidae	Lasioglossum
	Halictidae	Seladonia
	Megachilidae	Osmia
	Sphecidae	Sphecidae

	Bombyliidae	*Bombylius*
	Bombyliidae	*Parageron*
	Bombyliidae	*Usia*
	Empididae	*Empis*
	Syrphidae	*Chrysotoxum*
	Syrphidae	*Eristalis*
	Syrphidae	*Eupeodes*
	Syrphidae	*Platycheirus*
	Syrphidae	*Scaeva*
	Syrphidae	*Sphaerophoria*
	Syrphidae	“Syrphidae 1”
	Unknown	“Diptera 1”
	Tenthredinidae	Tenthredinidae
	unknown	Hymenoptera
Lepidoptera	Adelidae	Adelidae
	Hesperiidae	Thymelicus
	Pieridae	Colias
	Pieridae	Pieris
	Sphingidae	Macroglossum
	Unknown	Lepidoptera
Orthoptera	Unknown	Orthoptera 1

Table S10.- Preference of flower visitors for flower morphs. The table shows the probability of a visit to *L. narbonense* being to a L-morph flower. p-values result from exact binomial tests.

flower visitor	N	probability of visit a L-morph flower	p-value
Anthophora	12	0.500 [0.211 – 0.789]	1
Bombylius	23	0.348 [0.168 – 0.573]	0.210
Halictus	21	0.571 [0.340 – 0.782]	0.664
Lasioglossum	20	0.450 [0.231 – 0.685]	0.824
Malachius	44	0.500 [0.347 – 0.654]	1.000
Parageron	22	0.455 [0.244 – 0.678]	0.832
Usia	319	0.508 [0.452 – 0.564]	0.823

SUPPLEMENTARY TABLES FOR THE POLLINATION SUCCESS

Table S11.- Quantity component of pollination success. For each sampling the table displays the observed proportion of pollinated stigmas and the pollen load at each flower morph as well as the post-hoc comparisons between flower morphs. For the proportion of pollinated stigmas contrasts refer to the Odds Ratio (OR) while for the pollen load it refers to the Incidence Rate Ratio (IRR).

sampling	flower morph	proportion of pollinated stigmas				pollen load			
		value	contrasts			value	contrasts		
			OR (S / L)	z	p-value		IRR (S / L)	z	p-value
Caz-A-1	L-morph	0.90	5.21	2.233	0.026	19.99 [1.00-51.27]	2.89	4.084	<0.001
	S-morph	0.98				54.27 [5.60-124.40]			
Caz-B-1	L-morph	0.96	0.56	-0.713	0.476	26.02 [5.00-58.95]	1.32	0.777	0.437
	S-morph	0.92				38.27 [2.40-101.10]			
Caz-C-1	L-morph	0.90	1.50	0.866	0.387	24.14 [1.00-80.35]	1.18	0.585	0.559
	S-morph	0.93				21.22 [1.00-72.57]			
Hue-A-1	L-morph	0.99	3.14	0.694	0.487	52.1 [3.00-108.00]	1.21	0.776	0.437
	S-morph	1.00				64.23 [5.00-125.00]			
Hue-A-2	L-morph	0.97	2.29	0.835	0.404	88.56 [3.00-214.00]	0.78	-0.878	0.380
	S-morph	0.99				69.49 [2.7.00-180.10]			
Hue-A-3	L-morph	0.98	6.87	1.285	0.199	46.61 [2.00-162.60]	2.01	3.288	0.001
	S-morph	1.00				76.33 [6.65-178.05]			
Hue-B-1	L-morph	0.84	9.16	1.490	0.136	13.41 [1.00-94.00]	4.00	2.709	0.007
	S-morph	1.00				22.74 [5.00-71.30]			
Hue-C-1	L-morph	0.95	0.53	-1.139	0.255	39.45 [1.27-120.72]	0.64	-1.392	0.164
	S-morph	0.90				27.29 [1.00-74.12]			
Hue-D-1	L-morph	0.96	2.94	1.427	0.154	16.53 [1.00-65.83]	3.63	5.702	<0.001
	S-morph	0.99				54.28 [4.00-161.07]			
Nev-A-1	L-morph	0.83	2.90	2.998	0.003	20.96 [2.00-76.40]	1.68	2.306	0.021
	S-morph	0.94				37.26 [1.75-143.25]			
Nev-A-3	L-morph	0.93	4.37	1.617	0.106	21.31 [1.00-76.20]	2.36	2.417	0.016
	S-morph	0.99				39.42 [1.95-104.45]			
Nev-B-1	L-morph	1.00	0.73	-0.156	0.876	45.92 [2.00-147.10]	1.81	2.691	0.007
	S-morph	1.00				66.09 [7.22-150.77]			
Nev-B-3	L-morph	1.00	1.54	0.211	0.833	72.46 [31.85-123.45]	1.07	0.535	0.593
	S-morph	1.00				78.19 [15.93-158.33]			
Nev-C-1	L-morph	0.95	0.50	-1.107	0.268	19.91 [1.00-75.25]	1.59	1.241	0.215
	S-morph	0.90				28.48 [2.00-113.82]			
Nev-D-1	L-morph	0.96	0.83	-0.344	0.731	21.12 [1.97-79.23]	3.02	4.394	<0.001
	S-morph	0.95				74.16 [1.00-270.00]			
Nev-E-1	L-morph	1.00	0.10	-1.535	0.125	60.69 [4.00-168.20]	1.94	2.695	0.007
	S-morph	0.97				104.69 [14.88-263.62]			

Model used for the proportion of pollinated stigmas:

brglm(pollen.on.stigma ~ flower.morph, data = data, family = binomial).

Model used for the pollen load:

glmer(pollen.load ~ flower.morph + (1 | flower.id), data = data, family = poisson)

Table S12.- Quality component of pollination success. Pollen grain-tube relationship. The table display the relationship between pollen grains and pollen tubes at each flower morph and sampling as well as for the null model denoting such relationship under legitimate pollination. b1 relates to such relationship before stigma saturation. For those cases where such saturation existed, the number of pollen grains at which stigma saturated (c) and the slope of the relationship after such saturation (b2) are displayed. Post-hoc contrasts between flower morphs and between each flower morph and the corresponding null model are displayed.

sampling	flower morph	model			contrasts between morphs (L – S-morph flowers)			contrasts with the null model (open pollination – null model)		
		b1	b2	c	estimate	t ratio	p-value	estimate	t ratio	p-value
null models										
	L-morph	0.208	-0.003	54.27	0.023	1.267	0.206			
	S-morph	0.176	0.025	77.00						
samplings										
Caz-A-1	L-morph	0.327			0.191	6.003	<0.001	0.089	2.969	0.017
	S-morph	0.103	-0.019	60.00				-0.079	-3.94	0.001
Caz-B-1	L-morph	0.350			0.244	6.638	<0.001	0.124	3.505	0.003
	S-morph	0.080						-0.098	-4.607	<0.001
Caz-C-1	L-morph	0.392	0.102	45.94	0.062	1.398	0.502	0.157	4.688	<0.001
	S-morph	0.238	-0.047	36.00				0.118	3.45	0.004
Hue-A-1	L-morph	0.186			0.035	1.774	0.288	0.009	0.376	0.982
	S-morph	0.171						-0.003	-0.194	0.997
Hue-A-2	L-morph	0.223	0.022	67.63	0.08	3.347	0.005	0.031	1.261	0.588
	S-morph	0.147	0.003	107.00				-0.025	-1.506	0.435
Hue-A-3	L-morph	0.466	0.074	33.00	0.308	8.105	<0.001	0.239	5.763	<0.001
	S-morph	0.126						-0.046	-3.265	0.006
Hue-B-1	L-morph	0.274	-0.080	43.00	0.262	4.835	<0.001	0.088	1.979	0.198
	S-morph	0.019						-0.152	-4.264	<0.001
Hue-C-1	L-morph	0.229	0.089	43.81	0.176	4.969	<0.001	0.048	1.409	0.495
	S-morph	0.060						-0.105	-5.379	<0.001
Hue-D-1	L-morph	0.313	0.105	32.01	0.189	3.358	0.005	0.115	3.008	0.015
	S-morph	0.135	0.011	25.00				-0.051	-1.151	0.658
Nev-A-1	L-morph	0.178			0.17	10.473	<0.001	-0.013	-0.668	0.909
	S-morph	0.019						-0.161	-13.183	<0.001
Nev-A-3	L-morph	0.104			0.08	4.418	<0.001	-0.091	-4.634	<0.001
	S-morph	0.025						-0.148	-9.812	<0.001
Nev-B-1	L-morph	0.313			0.246	7.039	<0.001	0.097	2.6	0.048
	S-morph	0.049						-0.126	-9.71	<0.001
Nev-B-3	L-morph	0.114			0.049	2.764	0.031	-0.017	-0.777	0.865
	S-morph	0.129						-0.043	-2.87	0.023
Nev-C-1	L-morph	0.043			0.013	0.499	0.959	-0.155	-5.819	<0.001
	S-morph	0.029						-0.146	-8.54	<0.001
Nev-D-1	L-morph	0.159			0.099	5.733	<0.001	-0.038	-1.977	0.198
	S-morph	0.057	-0.014	96.00				-0.114	-7.986	<0.001
Nev-E-1	L-morph	0.211	-0.031	84.04	0.164	9.928	<0.001	0.01	0.436	0.972
	S-morph	0.041						-0.132	-10.992	<0.001

Model used for the pollen grain-tube relationship:

lmer(tubes ~ 0 + pollen.load * flower.morph * sampling + (1 | flower.id), data = data, na.action = na.omit)
